# The newly assembled chloroplast genome of *Aeluropus littoralis*: molecular feature characterization and phylogenetic analysis with related species

**DOI:** 10.1038/s41598-024-57141-8

**Published:** 2024-03-18

**Authors:** Walid Ben Romdhane, Abdullah Al-Doss, Afif Hassairi

**Affiliations:** College of Food and Agricultural Sciences, Plant Production Department, King Saud University, P.O. Box 2460, 11451 Riyadh, Saudi Arabia

**Keywords:** Comparative genomics, DNA sequencing, Next-generation sequencing, Phylogenomics, Genetic markers

## Abstract

*Aeluropus littoralis*, a halophyte grass, is widely distributed from the Mediterranean to the Indian subcontinent through the Mongolian Gobi. This model halophyte has garnered increasing attention owing to its use as forage and its high tolerance to environmental stressors. The chloroplast genomes of many plants have been extensively examined for molecular, phylogenetic and transplastomic applications. However, no published research on the *A. littoralis* chloroplast (cp) genome was discovered. Here, the entire chloroplast genome of *A. littoralis* was assembled implementing accurate long-read sequences. The entire chloroplast genome, with an estimated length of 135,532 bp (GC content: 38.2%), has a quadripartite architecture and includes a pair of inverted repeat (IR) regions, IRa and IRb (21,012 bp each), separated by a large and a small single-copy regions (80,823 and 12,685 bp, respectively). The features of *A. littoralis* consist of 133 genes that synthesize 87 peptides, 38 transfer RNAs, and 8 ribosomal RNAs. Of these genes, 86 were unique, whereas 19 were duplicated in IR regions. Additionally, a total of forty-six simple sequence repeats, categorized into 32-mono, four-di, two-tri, and eight-tetranucleotides, were discovered. Furthermore, ten sets of repeats greater than 20 bp were located primarily in the LSC region. Evolutionary analysis based on chloroplast sequence data revealed that *A. littoralis* with *A. lagopoides* and *A. sinensis* belong to the Aeluropodinae subtribe, which is a sister to the Eleusininae in the tribe Cynodonteae and the subfamily Chloridoideae. This subfamily belongs to the PACMAD clade, which contains the majority of the C4 photosynthetic plants in the Poaceae. The newly constructed *A. littoralis* cp genome offers valuable knowledge for DNA barcoding, phylogenetic, transplastomic research, and other biological studies.

## Introduction

Chloroplasts, tiny organelles found only in photosynthetic eukaryotic cells^1,2^, are unique because they have their own DNA and ribosomes^3^. Aside from their photosynthetic function, chloroplasts play an essential role in the biosynthesis of fatty acids, starch, and several amino acids^4,5^. The first complete chloroplast (cp) genome sequence was reported by Ohyama^6^ for the common liverwort species *Marchantia polymorpha*, followed by that for the tobacco plant *Nicotiana tabacum*^7^. To date, large numbers of chloroplast genomes have been sequenced, examined, and deposited in the NCBI organelle genome database (https://www.ncbi.nlm.nih.gov/genome/browse#!/organelles/); an expected rise in number as researchers exploit cutting-edge NGS technologies. In general, the chloroplast genome is circular and contains several genes vital for the maintenance of organelle and its functions, as well as those encoding ribosomal and transfer RNA^1,3,8^. The circular chloroplast genome of terrestrial plants is approximately 120–180 bp long^9^, with quadripartite features consisting of two inverted repeat regions (IR) separated by large (LSC) and small single copy (SSC) region^1,10,11^.

Poaceae is a large family of monocotyledons that are commonly known as grasses and are of particular interest to humans and animals. In recent decades, the picture of the evolutionary history of the grass family has developed using different techniques: restriction site maps of the chloroplast genome; sequences of the chloroplast genes (such as ndhF, rpoC2, rbcL, matK, and rps4); and sequences of several nuclear genes (such as phytochrome B and granule-bound starch synthase), sequences of nuclear ribosomal DNA (ITS), and ribosomal RNA sequences (18S rDNA)^12^. Molecular phylogenetic analyses have facilitated the division of the Poaceae family into 12 subfamilies, including three early-divergent small subfamilies, Anomochlooideae, Puelioideae, and Pharoideae, which include 4, 11 and 12 species, respectively^13^. The remaining nine subfamilies form two large sister clades: the PACMAD clade, which contains six subfamilies (Panicoideae, Arundinoideae, Chloridoideae, Micrairoideae, Aristidoideae, and Danthonioideae), and the BEP clade (synonym: BOP), which contains three subfamilies (Bambusoideae, Oryzoideae (synonym: Ehrhartoideae) and Pooideae)^12–17^. It has been reported that the C4 photosynthesis pathway has evolved 22 to 24 times in grasses, and it exists only in the PACMAD clade, whereas the BEP (BOP) clade contains only C3 taxa^18^.

The C4 plant *Aeluropus littoralis* is a perennial plant belonging to the Poaceae family, the Chloridoideae subfamily, and the Cynodonteae tribe^19^. *Aeluropus littoralis* is a monocotyledonous halophyte grass that processes salt glands and performs C4-type photosynthesis. This long stoloniferous grass species often has rooting stems^19,20^ and leaves that are close, short, stiff, flat and pointed at the top. The plant can withstand salt (NaCl) concentrations of up to 600 mM^19,21^ and is also considered drought and heat tolerant. It undergoes vegetative reproduction via its rhizomes and can also produce seeds^19,20,22^. Owing to these characteristics, *A. littoralis* can serve as a natural forage grass, growing in salt marshes and arid soils^19,23,24^. The subfamily Chloridoideae, to which the *Aeluropus* genus belongs, is a monophyletic group within the PACMAD clade of grasses (chloridoid grasses), as shown by molecular phylogenetic studies^13,25^. This subfamily includes approximately 131–140 genera with 1400–1700 species, the majority of which can thrive in arid regions and marginal salty land^12,15,17^. The most recent classification based on chloroplast and ITS sequences revealed that the Chloridoideae subfamily is classified into five tribes: Centropodieae, Triraphideae, Eragrostideae, Zoysieae, and Cynodonteae^12,14,15,26^. This subfamily is an important group for studying the evolutionary transition from C3 to C4 photosynthesis in grasses since the majority of its species uses the C4 photosynthetic pathway^13^. The C4 grasses are known to be particularly tolerant to drought, salt, and high temperature. This tolerance allows them to colonize harsh habitats through a unique network of anatomical, physiological, and molecular adaptations related to water, temperature, salinity, and excess light stresses^16^. For this purpose, they are considered important reservoirs of genes and promoters to improve resilience to abiotic stresses in cereals^27^. With progress in sequencing techniques over the last decade, plastomes have been increasingly adopted in grass phylogenetic studies^28^. By analyzing 122 sequenced nuclear loci from 47 species and 56 housekeeping genes, it was shown that *Aeluropus pungens* and *Odyssea paucinervis* form an independent *Aeluropus* subclade^26^. The same results were reported using nuclear sequences for two species *(Aeluropus pungens* and *Odyssea paucinervis*), which were classified into an independent subtribe named Aluropodinae under the Cynodonteae tribe^16^. Additionally, a phylogenetic tree was generated from the combined plastid data *(rps16-trnK spacer, rps16 intron, rpoC2, rpl32-trnL spacer, ndhF, ndhA intron, ccsA)* and the nuclear region (ITS). The plastid data place the plant *Odyssea paucinervis* as a sister to *Neobouteloua paucirracemosa* in Dactylocteniinae^26^. However, when the nuclear ITS sequences were used, the same plant was placed as a sister to *Aeluropus* in Aeluropodinae^26^. Referring to results based on 111 complete plastomes, the genus *Aeluropus* belongs to the Chloridoideae subfamily, the Cynodonteae tribe and the subtribe Aeluropodinae^17^. These authors demonstrated that in Cynodonteae, Eleusininae and Aeluropodinae are the third diverged lineages^17^. In their work, the subtribe Aeluropodinae included only *Aeluropus lagopoides* and *Aeluropus sinensis*.

Several research teams have characterized the chloroplast genomes of various plants for molecular selection, DNA barcoding, phylogenetic determination, and transplastomic purposes^29–32^. However, no published data were found in the literature on the chloroplast genome of *A. littoralis*. In this work, for the first time, we reported the entire chloroplast genome of *A. littoralis*, which we assembled based on the sequences of HiFi reads generated by the PacBio sequencing platform. Additionally, we examined simple sequence repeats (SSRs) and provided an overview of its general characteristics, gene contents, and organization. Lastly, we assessed its phylogenetic linkage to other chloroplast genomes in Poaceae family members. Our research sheds valuable light on the structural diversity and evolutionary history of chloroplast genomes in this widely distributed family of grasses.

## Results

### Assembly of chloroplast genome

**Figure 1:**
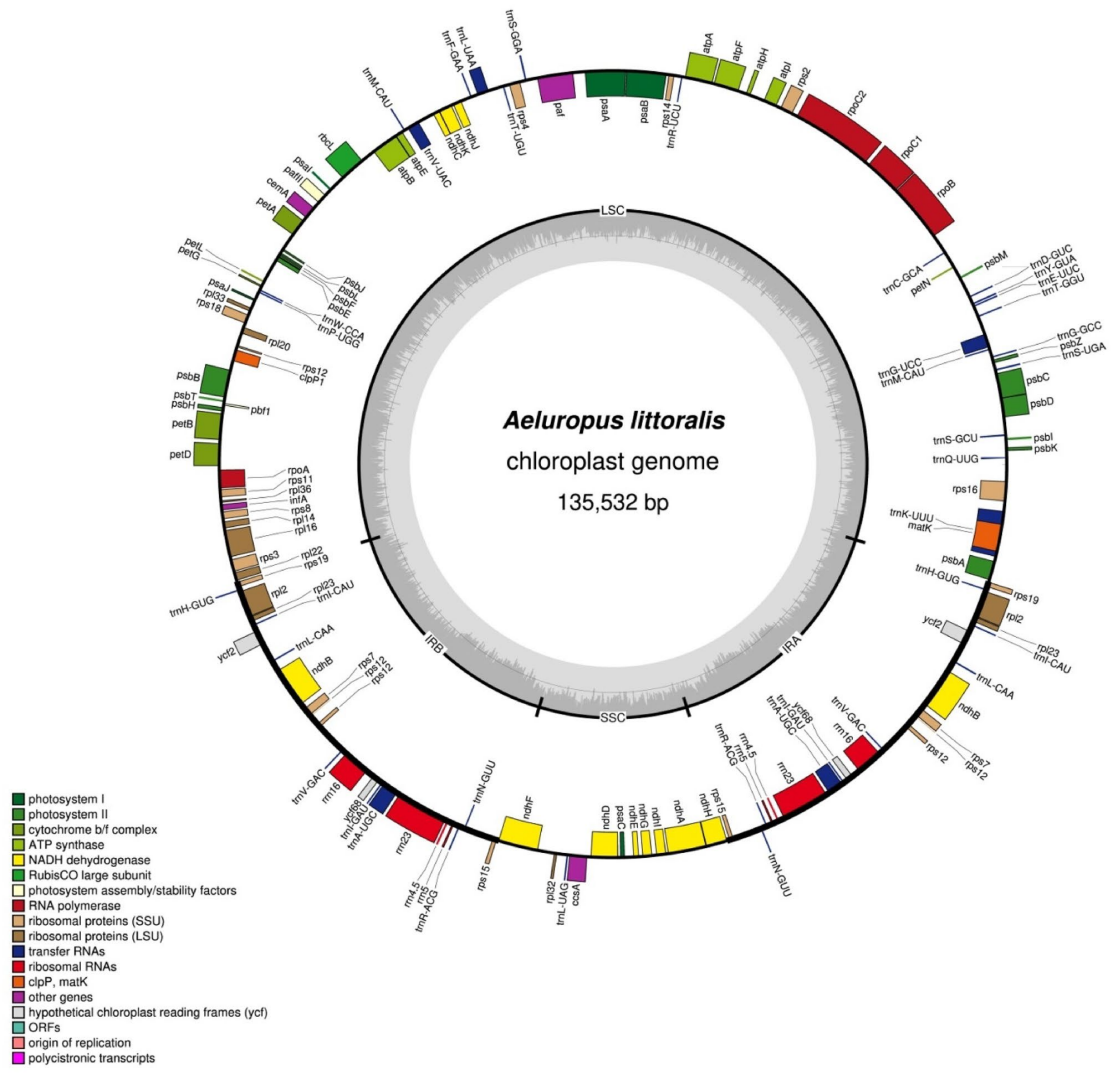
*A. littoralis* chloroplast genome map. Genes shown inside the circle are transcribed clockwise, whereas genes outside are transcribed counterclockwise. The light gray inner circle shows the AT content, the dark gray corresponds to the GC content.

The *A. littoralis* cp genome was assembled using selected chloroplast-related HiFi sequences obtained from the mapping of raw HiFi reads against a selected group of related cp genomes. The filtered 6907 reads with a mean length of 17,935 bp and a maximum length of 37,947 bp, accounting for 31,327,386 bp and ~ X230 coverage, were employed as input data for the cp genome assembly. The resulting 135,532 bp in length of *A. littoralis* cp genome with 38.2% GC content displayed a regular quadripartite structure architecture (Fig. 1), including an LSC of 80,823 bp, an SSC of 12,685 bp, and a pair of IRs of 21,012 bp each (Table 1). In addition, mapping of the HiFi long reads revealed that the *A. littoralis* cp genome exhibited two haplotypes, which differed in the 5’-3’ orientation of the SSC region and had an abundance ratio closer to 1:1. Indeed, a total of 1268 and 1076 long-reads were mapped to haplotype A (with a frequency of 0.54) and to haplotype B (with a frequency of 0.46), respectively (Fig. S1). The references of all reads mapped to either haplotype A or B were reported in Supplementary Table S1.

**Table 1:** Summary of the *A. littoralis* complete chloroplast genome characteristics.

*A. littoralis* cp genome characteristics	
Genome size	135,532 bp
LSC length	80,823 bp
SSC length	12,685 bp
IR length	21,012
GC content	38.2%
Total no. of genes	133
Protein-coding genes	87
tRNA	38
rRNA	8

### Chloroplast genome annotation

*A. littoralis* cp genome annotation using the Chloe annotation package determined the presence of genes encoding for: 8 ribosomal RNA (rRNA), 38 transfer RNA (tRNA), and 87 different proteins (Table 2). An in-depth look at the 133 genes revealed that 46 of them are implicated in the photosynthesis process, including the *rbcL* gene encoding for the Rubisco large subunit and *ndhA*—*K* genes encoding NADPH dehydrogenase proteins. Additionally, the *A. littoralis* cp genome included 31 genes encoding for RNA polymerase subunits and ribosomal proteins and 46 genes (tRNA + rRNA genes) involved in transcription and translation processes. In addition, 10 genes were implicated in several functions, such as cytochrome synthesis, carbon metabolism, proteolysis, and RNA processing. Interestingly, a gene structure analysis indicated that 112 genes were intronless, while 21 annotated genes had introns; 19 of these genes harbored a single intron, and only 2, *rps12* and *PafI*, contained 2 introns each (Supplementary Table S2).

**Table 2:** Functional gene groups in *A. littoralis* complete cp genome.

Category	Group of genes	Name of genes	Number
Photosynthesis	Subunits of ATP synthase	*atpA, atpB, atpE, atpF, atpH, atpI*	6
	Subunits of photosystem I	*psaA, psaB, psaC, psaI, psaJ*	5
	Subunits of photosystem II	*pafI, pbf1, psbA, psbB, psbC, psbD, psbE, psbF, psbH, psbI, psbJ, psbK, psbL, psbM, psbT, psbZ,*	16
	Subunits of NADH-dehydrogenase	*ndhA, ndhB, ndhB, ndhC, ndhD, ndhE, ndhF, ndhG, ndhH, ndhI, ndhJ, ndhK*	12
	Subunits of cytochrome b/f complex	*petA, petB, petD, petG, petL, petN*	6
	Subunit of rubisco	*rbcL*	1
Replication	Large subunit of ribosome	*rpl2, rpl2, rpl14, rpl16, rpl20, rpl22, rpl23, rpl23, rpl32, rpl33, rpl36*	11
	DNA dependent RNA polymerase	*rpoA, rpoB, rpoC1, rpoC2*	4
	Small subunit of ribosome	*rps2, rps3, rps4, rps7, rps7, rps8, rps11, rps12, rps12, rps14, rps15, rps15, rps16, rps18, rps19, rps19*	16
Other genes	Cytochrom synthesis	*ccsA*	1
	Envelop membrane	*cemA*	1
	Protease	*clpP1*	1
	Translational initiation factor	*infA*	1
	Maturase	*matK*	1
	Unkown function	*ycf2,ycf2, pafII(ycf4), ycf68, ycf68*	5

### Repeat sequence surveys

**Figure 2:**
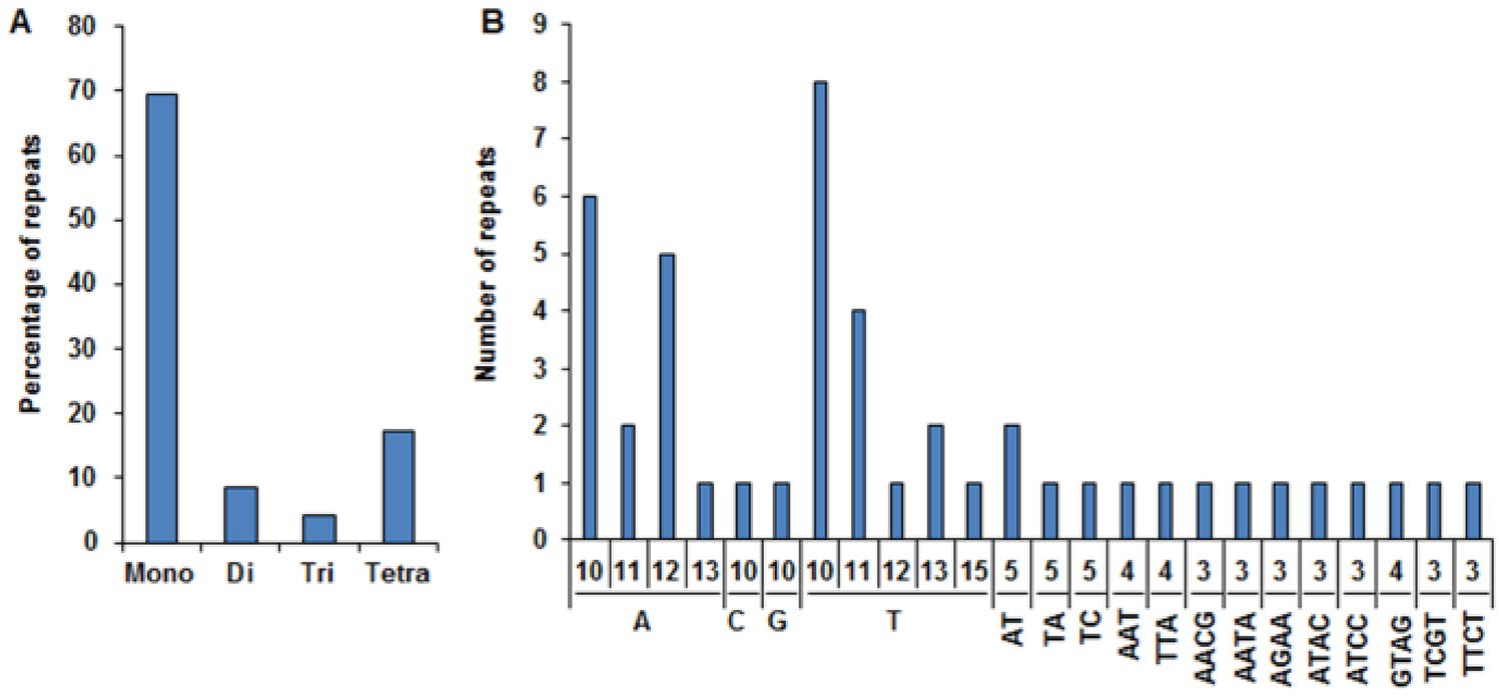
Simple sequence repeats (SSR) in the *A. littoralis* cp genome. (**A**) Frequency of identified SSR types. (**B**) Number of different identified SSR motifs.

In *A. littoralis* cp genome, a full set of 46 simple sequence repeats (SSRs) were discovered. Among them, 69.56% (n = 32) were mononucleotide repeats composed of either A or T (Fig. 2). No penta- or hexa-nucleotide SSRs were detected in the *A. littoralis* cp genome. Interestingly, the identified SSRs were largely abundant in LSC, with a frequency of 78.26%, compared with those in SSC and IRs. Our repeat search identified 10 sets of repeats longer than 20 bp from the chloroplast genome of *A. littoralis*. The length of the repeats ranged between 20 and 67 bp. The majority of the repeats were in the LSC region, except for one in the SSC region. Seven of them were in intergenic spacers, two were in *rpoC2*, and one was in *rps18* gene (Supplementary Table S3).

### Putative RNA editing site analysis

RNA editing is pivotal post-transcriptional regulatory process of cp-genes expression through nucleotide insertions, deletions, and substitutions^1^. By examining *A. littoralis* cp sequence, 78 RNA editing sites were predicted, involving 31 protein-coding genes. Remarkably, 31% of the predicted RNA editing sites were noticed within the *ndh* genes (ndhA [6], ndhB [7], ndhD [2], ndhF [5], ndhG [1], ndhH [2], and ndhK [1]; however, the rpoC2 gene had the largest number of predicted RNA editing sites [12], followed by matK [9], ndhB [7], ndhA [6], rpoB and ndhF [5 each], cemA [3], and atpA, *ndhD*, *ndhH*, *rpl23*, *rps18*, *rps19*, and *ycf3* (2 each), whereas the other 17 genes had only one predicted editing site. All the predicted RNA editing sites involved the conversion of cytosine (C) to uracil (U), which may have caused amino acid changes. A major portion (76%) of the predicted RNA editing occurred in the second codon, and only 24% occurred in the first position of the codon (Supplementary Table S4).

### Codon usage

**Figure 3:**
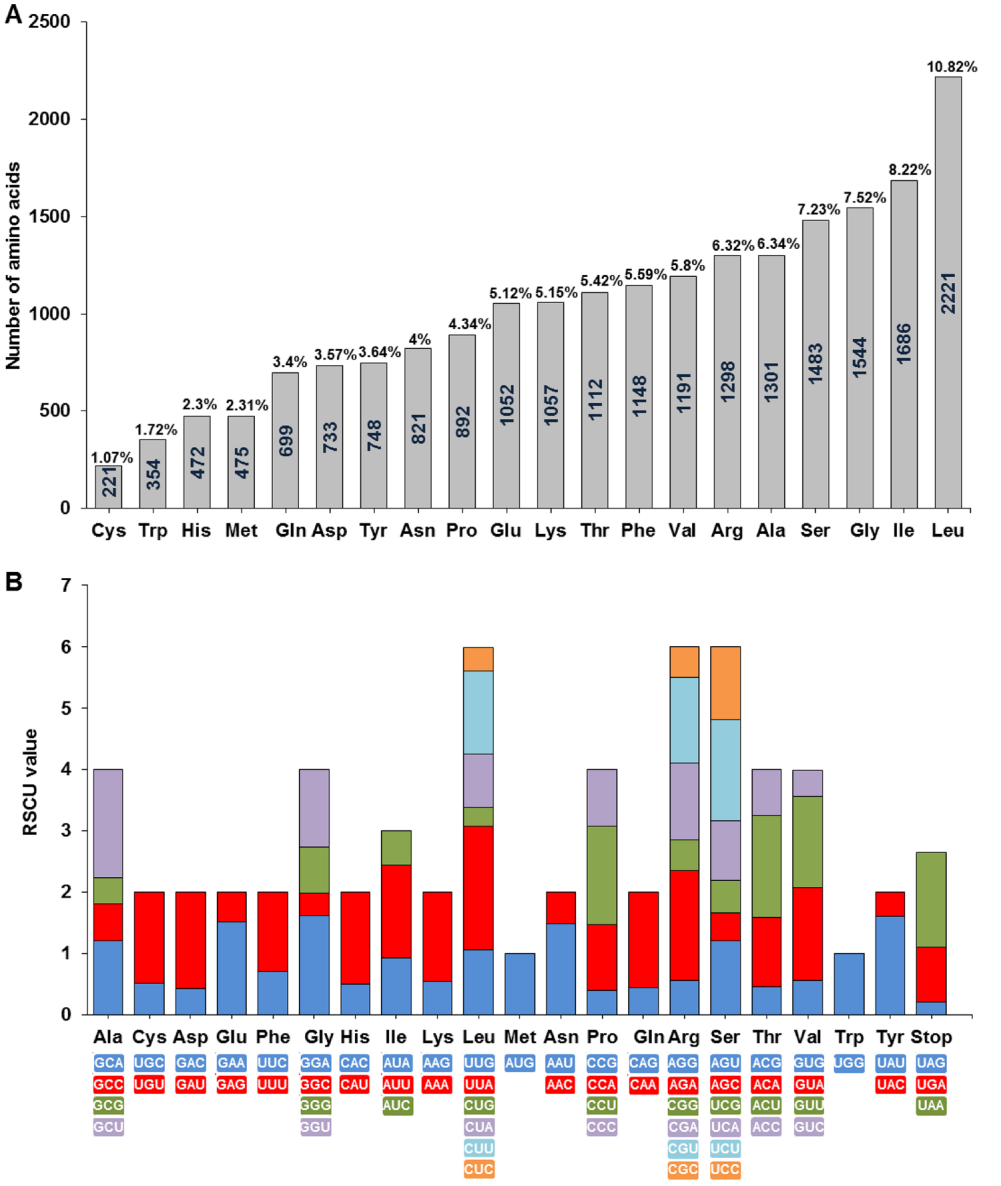
Codon usage patterns analysis of the *A. littoralis* chloroplast genome. (**A**) Frequency analysis of amino-acids in *A. littoralis* cp protein-coding genes. (**B**) RSCU values of 20 amino acid and stop codons in all protein-coding genes of the *A. littoralis* cp genome*.*

The sequences of the 87 protein-coding genes were retrieved from the *A. littoralis* cp genome, and the codon number and codon usage frequency were evaluated. A total of 20,508 different codons were analyzed among the 87 protein-coding genes. The nucleotide triplet (AUU), which encodes the amino acid isoleucine, was the most abundant, with an average number of 847, while the UGC triplet, which encodes cysteine, was the least abundant (56), except for the stop codons (Fig. 3). Among the 20 amino acids, leucine, isoleucine, glycine, and serine were the most abundant, with 2221 (10.82%), 1686 (8.22%), 1544 (7.52%), and 1482 (7.23%) codons, respectively; in addition, the rarest one was cysteine, with 221 (1.07%) codons. To identify codon usage profiles in the *A. littoralis* cp genome, the average relative synonymous codon usage (RSCU) values were estimated (Fig. 3). The look at these RSCU values revealed that thirty codons were most frequently used (RSCU > 1), whereas thirty-two codons showed little usage (RSCU < 1). Contrary, the AUG (methionine) and UGG (tryptophan) codons showed a lack of bias (RSCU = 1). Interestingly, within the codons with RSCU > 1, twenty-four were enriched in A/U, 12 (40%) ended in A, and 18 (60%) ended in U, suggesting that A/T nucleotide bases are preferred at the third position of the codon in the *A .littoralis* cp genome.

### Comparisons of *Aeluropus* cp genomes boundary regions

**Figure 4:**
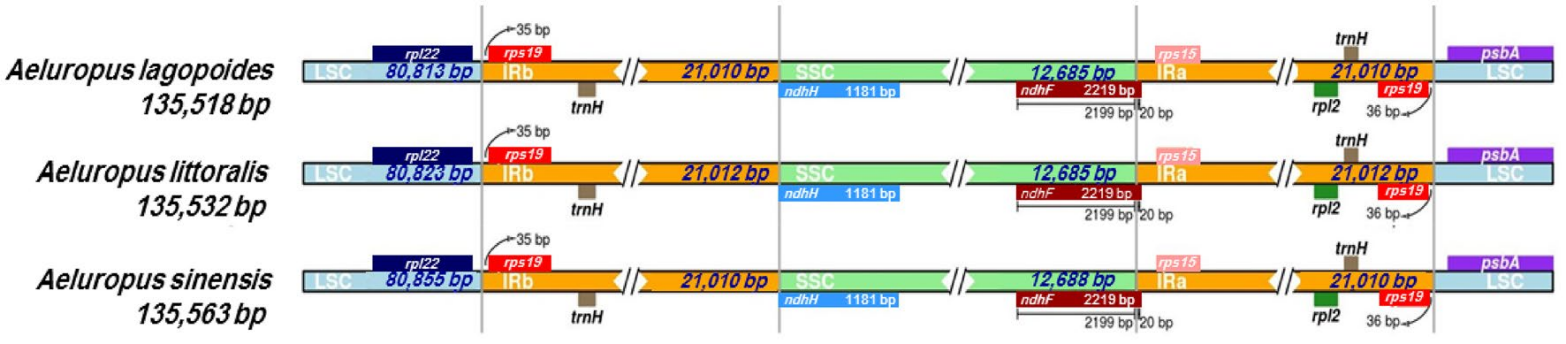
Comparison of the boundaries between LSC, SSC, and IR regions among the three *Aeluropus species* cp genomes.

To gain insight into the evolutionary history of the genus *Aeluropus*, the expansion and contraction variation in junction regions were monitored via the comparison of border genes and regions across the cp genomes of the genus *Aeluropus* (Fig. 4 and Table S5). As illustrated in Fig. 4 and Table S4, the cp genomes of the genus *Aeluropus* showed high identity in terms of gene order, gene number, as well as at their IRa/LSC and IRb/SSC boundary regions. The fragment size of *rpl22*-*rps19* positioned in the IRb region was 35 bp in all evaluated *Aeluropus* species cp genomes. IRa/LSC was located in intergenic regions between the *rps19* and *psbA* genes. The length of *rps19*-*psbA* was 36 bp in all cp genomes of the genus *Aeluropus*. The IRb/SSC junctions were enclosed in the *ndhF* gene, and this gene was prolonged by 20 bp in the IRb region. The *ndhH* gene crossed the SSC/IRa region in all the cp genomes of the genus *Aeluropus*. Although the IRa, IRb, and SSC regions were conserved in all cp genomes of the genus *Aeluropus,* slight differences in LSC regions in term of length were revealed (Fig. 4).

**Figure 5:**
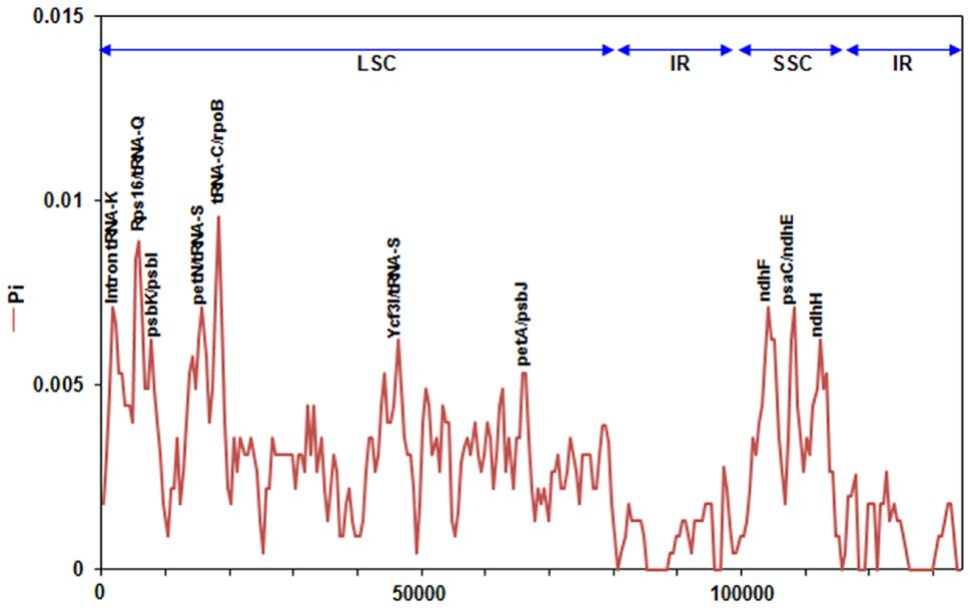
Nucleotide divergence analysis across *Aeluropus species* cp genomes.

The divergence hotspots between the three *Aeluropus* species cp genomes were computed through nucleotide diversity analysis using DnaSP software. As shown in Fig. 5, the nucleotide diversity index (Pi) ranged from 0 to 0.0088 with an average value of 0.0031. A greater number of genetic diversity hotspots were revealed in the LSC region with seven hotspots; however, three hotspots were located in the SSC region. The greatest genetic diversity was located in *Rps16*-*tRNA-Q* gene junctions and *tRNA-C*-*rpoB* gene junctions with Pi = 0.00889 and Pi = 0.00884, respectively. The IR region had the lowest Pi values, which suggested that it was more conserved than the LSC and SSC regions across the *Aeluropus* species cp genomes.

### Phylogenetic analysis

**Figure 6:**
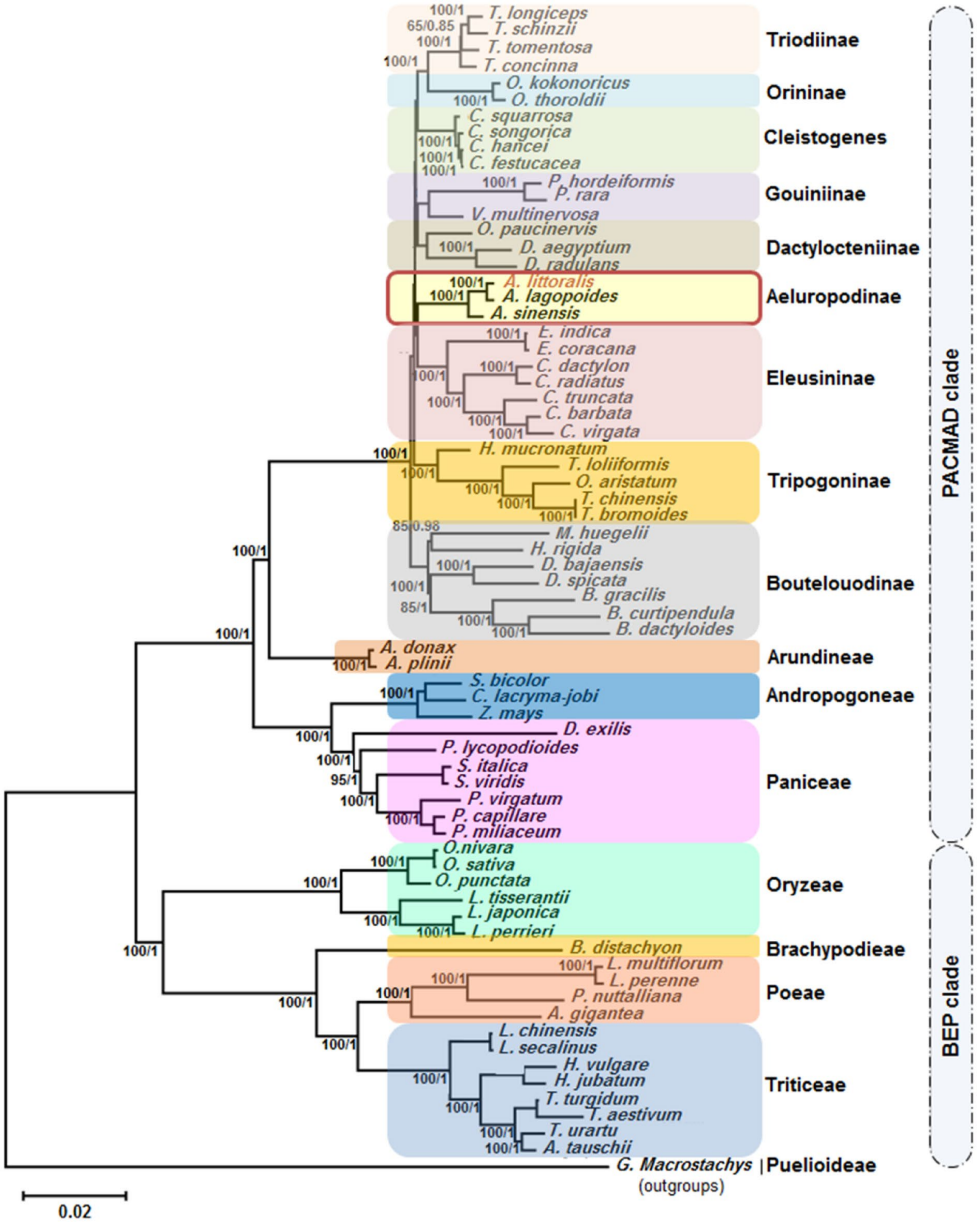
Maximum likelihood and Bayesian inference phylogenetic tree based on complete cp genomes of *A. littoralis* and related-species within the Poaceae family. Bootstrap and posterior probability support values are indicated above each node.

To uncover more about evolution and phylogenetic positions of *A. littoralis* a maximum likelihood and Bayesian inference phylogenetic tree with 1000 bootstrap replicates was built using complete cp genomes (Fig. 6) as well as shared amino-acid protein sequences (Fig. S2). These trees regroup *A. littoralis* and its related members among the Poaceae family, including *A. lagopoides*, *A. sinensis*, *O. sativa*, *S. italica*, *S. bicolor*, *P. nuttalliana*, *Z. mays*, *T. aestivum*, and *H. vulgare* (Fig. 6, Fig. S2). The two generated trees showed similar topologies. In addition, the selected species were subdivided into 16 groups, namely, Triodiinae, Orininae, Cleistogenes, Gouiniinae, Dactylocteniinae, Aeluropodinae, Eleusininae, Tripogoninae, Boutelouodinae, Arundineae, Andropogoneae, Paniceae, Oryzeae, Brachypodieae, Poeae, and Triticeae. The results highlighted that *A. littoralis*, *A. lagopoides*, and *A. sinensis* form a single subtribe, Aeluropodinae, within the Cynodonteae tribe from the Chloridoideae subfamily. The Aeluropodinae and Euleusininae subtribe are sister groups with bootstrap values of 100 and posterior probability values of 1 (Fig. 6). These two subtribes are the third diverged lineage in Cynodonteae. Thus, the species of Aeluropodinae subtribe were clustered with PACMAD species, which are distinguished by their C4 photosynthesis. Additionally, a total of twenty selected species were clustered into four sister groups composed of the tribes Oryzeae, Brachypodieae, Poeae, and Triticeae, which formed the BEP clade harboring species distinguished by their C3 photosynthesis, including *O. sativa*, *P. nuttalliana*, *B. distachyon*, *L. chinensis*, *T. aestivum*, and *H. vulgare*. The *Odyssea paucinervis* species was shown to be sister to *Dactylocterium aegyptium* and *D. radulans* species in the Dactylocteniinae subtribe and not in Aeluropodinae. Moreover, the *Eleusine coracana* and *Eleusine indica* species from the Euleusininae subtribe are the sisters nearest to *A. littoralis* (Fig. 6). Finally, outside the subfamily Chloridoideae, the species nearest to *A. littoralis* belong to the following tribes: Arundineae (Arundinoideae), Andropogoneae (Panicoideae), and Paniceae (Panicoideae). The *O. sativa*, *P. nuttalliana*, *B. distachyon*, *L. chinensis*, *T. aestivum*, and *H. vulgare* cp genomes, which belong to the BEP clade, exhibited remarkable diversity from the *A. littoralis* cp genome and formed a C3 photosynthesis-enriched cluster separate from the rest.

### Divergence time estimations

The time of divergence estimation was illustrated in Fig.S3. The nine tribes (Chloridoideae), Arundineae (Arundinoideae), Andropogoneae (Panicoideae), and Paniceae (Panicoideae) diverged from BEP clade the approximately 45 million years ago (Mya). Interestingly, the results showed that in Cynodonteae, Eleusininae and Aeluropodinae are the third diverged lineages from other seven tribes 23.2 Mya. In the Aeluropodinae subtribe, *A. littoralis* and *A. lagopoides* diverged from *A. sinesis* approximately 4.3 Mya (Fig.S3).

## Discussion

The genus *Aeluropus* consists of 6 species that are distributed mainly in saline habitats from the Mediterranean to the Indian subcontinent through the Mongolian Sahara^24^. *A. littoralis* is a perennial plant belonging to the *Aeluropus* genus from the Poaceae family of flowering plants^19^. Due to its small genome size, rapid growth rate, high tolerance to salt stress and multiple environmental stressors, high biomass production, and frequent forage use, *A. littoralis* is considered a model halophyte with increasing attention. The assembled *A. littoralis* chloroplast genome presents a common quadripartite structure and is similar in size to that of the majority of Poaceae species among angiosperms^1,33–39^. The *A. littoralis* cp genome comprises two IR regions (21,012 bp each) that are distanced by the LSC region (80,823 bp) and the SSC region (12,685 bp), indicating that the assembled cp sequence displayed full coverage with no abnormalities. Generally, the typical terrestrial plant cp genome size is 120 to 180 kb, with IR regions ranging from 10 to 30 kb^1,9^. The 38.24% GC content and this AT-rich feature of the *A. littoralis* cp genome are concordant with those reported for other plants, including 38.2% for *A. sinensis*, *A. lagopoides*^40^, *S. bicolor* (38.5%)^35^, *S. italica* (38.9%)^38^, *H. vulgare* (38.3%)^35^, *Z. mays* (38.5%)^33^, *G. hirsutum* (37.2%)^41^, and *A. thaliana* (36.3%)^42^. Interestingly, the *A. littoralis* cp genome was shown to be present under two chloroplast structural haplotypes based on long-read sequencing data assembly. These results are in agreement with those reported by Wang and Lanfear^43^, who confirmed the presence of two chloroplast structural haplotypes that occur with equal frequency in most land plant individuals.

Comprehensive analysis of the *A. littoralis* cp genome revealed that it contains coding regions (54.46% of the genome) harboring 133 genes, 87 of which are protein coding genes (44.59%), 8 are rRNA genes (6.77%), and 38 are tRNA genes (2.1%). Almost 85% of the cp-identified genes were intronless, 14% contained one intron, and *rps12* and *pafI* were the two genes with two introns each. These findings are in line with several cp-structures of angiosperm plants, which include 120–140 genes, 80–90 of which encode proteins, 30–40 of which encode transfer RNA genes, and 4–10 of which encode ribosomal RNA^1,44^. Likewise, similar *Setaria viridis* cp genome features were reported by Wang and Gao^37^. Thus, the cp genome features of land plants seem to be quite universal^45^. According to multitude studies, cp-SSR and tandem repeats are extremely variable DNA markers and are beneficial for diversity and population genetics analysis studies^46–49^. A total of 46 SSRs and 10 long repeats were noticed in the *A. littoralis* cp genome. Our findings were consistent with previous researches reporting that the common cp SSR markers identified were composed of A or T nucleotides and rarely included C and G nucleotides^41^. The identified cp SSRs and long tandem repeats could provide useful sequence resources for further molecular genetic studies of *A. littoralis*, including assessments of species genetic diversity and evolutionary studies.

RNA editing constitutes a common mechanism for cp gene expression modulation in plants through nucleotide insertions, deletions, and substitutions^50^. Our results indicated that the *A. littoralis* cp genome contains 78 predicted RNA editing sites dispersed among 31 protein-coding genes. All the predicted RNA editing sites resulted in the conversion of cytosine to uracil predominantly at the 2nd position of the codon. The predominant RNA editing type revealed in the *A. littoralis* cp genome was comparable to that observed in rice^51^, proso millet^52^, wheat^53^, and maize^33^. Intriguingly, cytosine—uracil conversion is the most common RNA editing type in plants^54^. Recently, Ramadan^55^ reported that differential RNA editing of the *ndhB* gene of the desert plant *Calotropis procera* led to the control of photosynthesis across different daylight periods. Moreover, owing to the involvement of chloroplast genes in photosynthesis and metabolite biosynthesis, cp gene expression appears to be crucial for plant responses to environmental stress^56^. The high number of predicted RNA editing sites in *A. littoralis* cp genome, particularly in important genes such as the *ndh* and *psb* genes, could be one of the keys to tolerance and the dynamic response to environmental stressors. Thus, it was recently reported that *Robinia pseudoacacia* chloroplastic development and PSI/PSII-related genes, including *ndhH*, *ndhE*, *psaA*, *psaB*, *psbA*, *psbD*, *psaC*, *psbC*, *ropA*, and *rps7*, are involved in the response to salinity.

The pattern of codon usage bias varies among species and between the genes within an organism^57^. Our results revealed that the AUU nucleotide triplet coding for the isoleucine amino acid was the most abundant while the UGC triplet that encodes cysteine was the least abundant. Thirty codons with RSCU > 1 were frequently used and thirty-two codons showed little usage. Except for methionine and tryptophan, which lack synonymous codons, all amino acids are represented by 2–6 synonymous codons. Twenty-four of the codons with RSCU values greater than one were rich in A/U, indicating that A/T nucleotide bases are preferred at the 3rd codon position in the *A. littoralis* cp genome. This high preference for A/T nucleotide at the 3rd codon position was similarly noted in numerous terrestrial plant cp genomes^1,58,59^. Additionally, Somaratne et al.^60^ pointed to similar codon usage patterns in several analyzed Poaceae cp genomes associated with AT-rich bias particularly in the third codon position.

A phylogenetic tree was built using the entire cp-genome as well as the shared protein sequences of *A. littoralis* and sixty-nine selected Poaceae species. The inferred phylogenetic tree clearly showed two large distinct clades: the BEP clade and the PACMAD clade. The *A. littoralis*, *A. logopoides*, and *A. sinensis* species form an independent subtribe, Aeluropodinae, in the Cynodonteae tribe of the Chloridoideae subfamily. On the other hand, *O. paucinervis*, *D. aegyptium*, and *D. radulans* were shown to be sister species in the Dactylocteniinae subtribe and not in Aeluropodinae. These results are in agreement with those reported by Peterson et al.^26^ and Wang et al.^17^, who used plastid sequences in their phylogenetic analyses. However, when nuclear sequences were used, *A. pungens* and *O. paucinervis* were classified into Aeluropodinae subtribe in the Cynodonteae tribe^16^. Our future work aims to sequence and assemble at chromosome-scale *A. littoralis* genome will help to clarify this issue. The divergence time estimation revealed that Aeluropodinae and Eleusininae are sister subtribes. This means that *E. coracana* and *E. indica* are the nearest species to *A. littoralis*. Moreover, the Aeluropodinae diverged 45 Mya from the subtribes with C4 plants of Andropogoneae (containing S. *bicolor* and *Z. mays*) and Paniceae (containing *Panicum capillare*, *Panicum lycopodioides*, *Panicum miliaceum*, *Panicum virgatum*, *Setaria italic*a and *Setaria viridis*). However, the four subtribes belonging to the BEP clade and specified by their C3 photosynthesis plants diverged 59.1 Mya earlier in the large Poaceae family. Our results were in accordance with previous phylogenetic relationships within Poaceae^17,35,37,38,40^.

## Conclusions

In this work, the entire cp genome sequence of *A. littoralis* was assembled using raw reads generated via PacBio HiFi read sequencing technology. The *A. littoralis* cp genome was 135,532 bp in length and had a common circular quadripartite structure. This cp genome encodes 133 genes, 85% of which are intronless, along with 64 codons that correspond to 20 amino acids, with the AUU and UGC codons being the most and the least abundant, respectively. Codon bias analysis revealed a marked preferential usage of codons containing A/U in the third position, particularly among those with RSCU values greater than 1. We also identified a total of 46 SSRs and 10 long repeats. A comparison of the *A. littoralis* cp genome with those of two other *Aeluropus* species confirmed a highly conserved structure and slight polymorphic spot regions. Phylogenetic analysis based on entire cp genomes demonstrated that *A. littoralis*, *A. lagopoides*, and *A. sinensis* form a single subtribe, Aeluropodinae, within the tribe of Cynodonteae from the subfamily Chloridoideae. The subtribes Aeluropodinae and Euleusininae are sister groups with bootstrap values of 100. These two subtribes are the third diverged lineage in Cynodonteae. Thus, *A. littoralis* is clustered with PACMAD species, which are mainly distinguished by their C4 photosynthesis. The findings from this study offer valuable genetic information and a framework for further phylogeographic, population genetics, and plastid genetic engineering research on *A. littoralis* and related species.

## Methods

### Plant materials and growth conditions

*Aeluropus littoralis* cuttings and seeds were collected from a salty area (25° 04′ 48.6″ N 46° 20′ 27.7″ E) in Salboukh region, located north of Riyadh, Saudi Arabia. The taxonomic identification was verified by Prof. Dr. Abdulaziz Assaeed, who is affiliated with College of Food and Agriculture Sciences, King Saud University; a specimen under voucher number 69,107 was placed in the herbarium of the college of food and agriculture sciences, King Saud University. *A. littoralis* cuttings derived from a single seed were rooted in sterile water and subsequently transplanted to a hydroponic system that used the nutritive solution detailed previously by Ben Romdhane et al.^61^. *A. littoralis* plants were grown in greenhouse conditions under a 16 h/8 h light/dark cycle. After 2 months, fresh tissues were harvested from *A. littoralis* plants and immediately ground into a fine powder in a mortar pre-cooled with liquid nitrogen. Tissue samples were then stored at − 80 °C prior to DNA extraction.

### DNA extraction, library preparation, and sequencing

The DNA extraction protocol used in this study was based on the conventional CTAB method^62^. An Epoch microplate spectrophotometer (BioTek, Winooski, VT, USA) was used to measure the gDNA concentration, and two distinct agarose gel concentrations (0.8% for 1 h at 70 mV and 0.6% for 15 h at 35 mV) were employed to examine the sample's quality. The HMW-gDNA was purified using AMPure PB beads (Pacific Biosciences) were employed to purify the HMW-gDNA, which was further eluted via PacBio elution buffer, and inspected for quality through an Agilent 2100 Bioanalyzer (Agilent).

Utilizing the HiFi protocol (PacBio), two libraries for single-molecule real-time (SMRT) sequencing were developed from the extracted gDNA. The whole-genome sequencing (WGS) of *A. littoralis* was conducted by the DNA Link Sequencing Lab (DNA Link Inc, Seoul, Republic of Korea).

### Genome compiling and gene labeling

The chloroplast-related reads were fished from WGS HiFi reads through their alignment to the closest cp-genomes [*Oryza sativa* (KM088016), *Sorghum bicolor* (NC-008602), *Setaria italica* (NC-022850), *Zea mays* (NC-001666), *Aeluropus logopoides* (NC_042858), *Brachypodium distachyon* (NC-011032), and *Puccinellia nuttalliana* (NC-027485)] via the Minimap2 aligner^63^. The cp-related reads were subsequently compiled using CLC genomics workbench V22.0 software.

The *A. littoralis* chloroplast sequence was annotated with the GeSeq pipeline^64^ using the Chloe V0.1.0 annotation package. The predicted annotation and the start/stop codon were manually inspected using BLAST against the Nr database. Genes encoding transfer RNA (tRNA) were assessed by using tRNAscan-SE 2.0 software with default settings^65^. The graphical map of the *A. littoralis* chloroplast genome was drawn by the Organellar Genome DRAW toolkit (https://chlorobox.mpimp-golm.mpg.de/OGDraw.html)^66^. The *A. littoralis* chloroplast genome sequence was deposited in the NCBI GenBank database with the accession number ON357749.

### Exploration of chloroplast genome repeats

The MISA tool was employed discover simple-sequence-repeats (SSRs) (https://webblast.ipk-gatersleben.de/misa/)^67^ with the following parameters: ten for mononucleotides, five for dinucleotides, four for trinucleotides and three for tetra, penta, and hexa-nucleotide SSR motifs.

Repeat sequences longer than 20 nucleotides were predicted by the tandem repeats finder program with the following parameters: (2, 7, 7) for alignment parameters (match, mismatch, indels), 80 for minimum alignment score to report repeat, and maximum period size of 500.

### Prediction of RNA editing sites

Prediction of putative RNA editing sites in the *A. littoralis* chloroplast genome was carried out using the plant RNA editing prepact tool (http://www.prepact.de/prepact-main.php). For predicting potential RNA editing sites, the *Z. mays* (NC_001666.2) full organelle complete record was fixed as a database for BLAST with an E-value cutoff of 0.8.

### Examination of codon use

The CodonW program (V1.4.4) was executed to examine the preferred synonymous codons for protein-coding genes and to examine RSCU values.

### Phylogenetic analysis

By employing the cp genomes and shared protein sequences of *A. littoralis* and sixty-nine *Poaceae* species (Supplementary Table S6), phylogenetic linkage was assessed. The *Guaduella macrostachys* chloroplast genome (NC_061343) belongs to the *Puelioideae* subfamily (used as outgroup). The MAFFT program (v7.520)^68^ was executed to compute the alignment of nucleic acid and protein sequences. The MEGA11 program^69^ was implemented to determine the best substitution model, and the GTR + G model was selected (Supplementary Table S7). Maximum likelihood analysis was conducted via the RAxML program^70^ (v8.2.11) with 1000 bootstrap replicates and the GTRGAMMA model. Bayesian inference analysis was carried out in the MrBayes program^71^ (v3.2.6) with Markov chain Monte Carlo (MCMC) runs for 1,000,000 generations with a random starting tree, and one tree was sampled every 1000 steps. The first 25% of steps were discarded as burn-in.

### Divergence time estimations

The divergence time was estimated for each internal node of the generated phylogenetic tree using MEGA11^69^. The RelTime method was utilized in dating analyses via calibration of the node time to fine-tune the molecular clock. The maximum age of *Triodia longiceps* and *Aegilops tauschii* nodes was assigned as 51.9 million years ago (Mya). The minimum age of the *Triodia longiceps* and *Aegilops tauschii* nodes was assigned as 41.4 Mya. The minimum and maximum ages of the *Oryza sativa* and *Aegilops tauschii* nodes were pointed as 41.5‒62 Mya, respectively.

### Ethical approval and consent to participate

The authors have respected the relevant institutional, national and international guidelines in collecting biological materials for this work. This research contributes to facilitating future studies in species identification, phylogeny, and transplastomic research.

### Supplementary Information

Supplementary Figures.

Supplementary Table S1.

Supplementary Tables.

## Data Availability

The *A. littoralis* cp genome was deposited into NCBI database (ON357749). The PacBio sequencing reads utilized during the study are available in the SRA (Sequence Read Archive) of NCBI under the accession number PRJNA1075656.
