# Supplementary material for: The newly assembled chloroplast genome of Aeluropus littoralis: molecular feature characterization and phylogenetic analysis with related species
Source: Sci Rep. 2024 Mar 18;14:6472. doi: 10.1038/s41598-024-57141-8 (PMC10948853; doi:10.1038/s41598-024-57141-8)
Supplement: Supplementary file 1 — Supplementary Figures. [file 41598_2024_57141_MOESM1_ESM.docx]

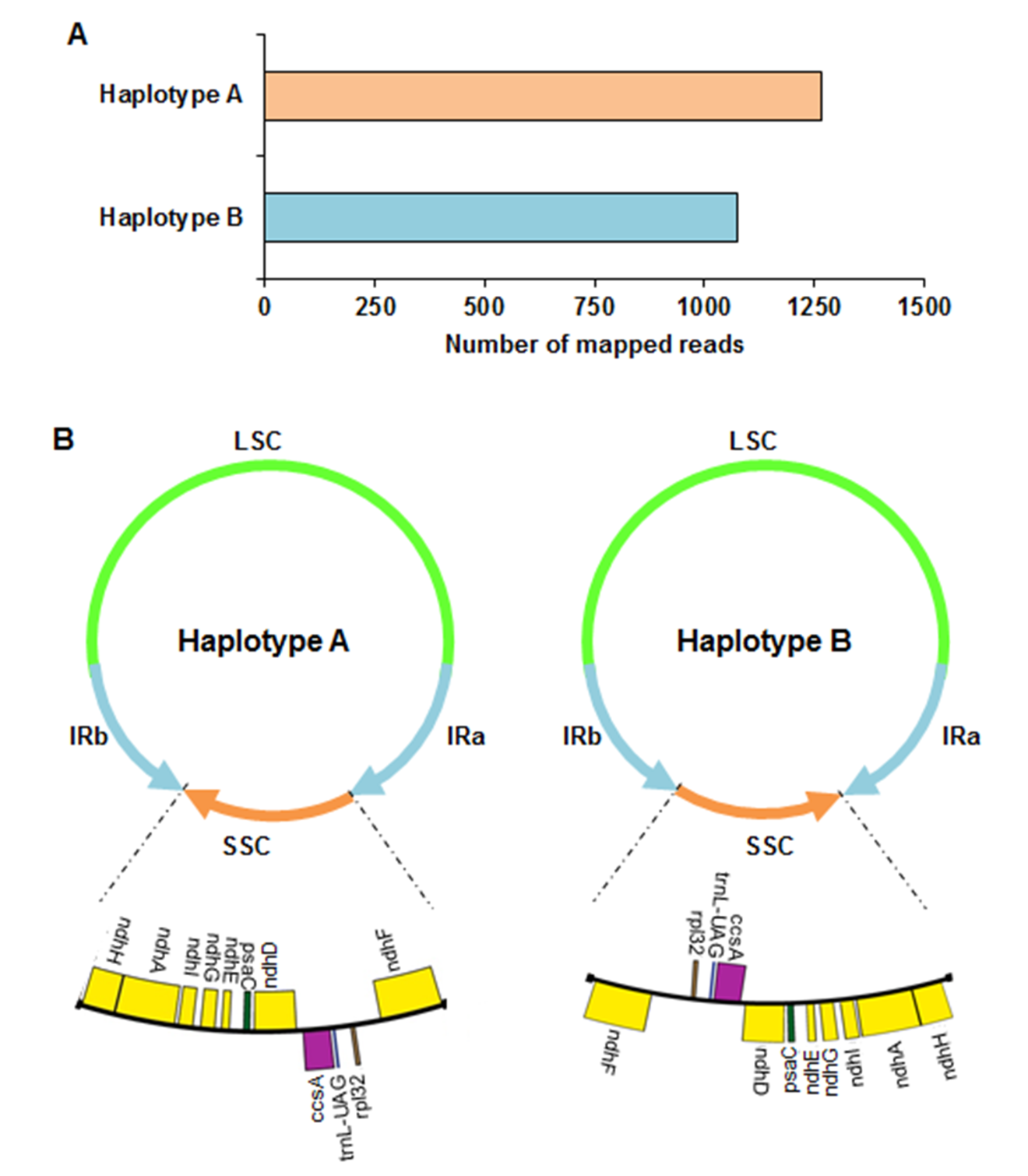


**Figure S1.** Structures of *A. littoralis* cp genome haplotypes. **(A)** The number of long-reads mapped to either haplotype A or haplotype B. **(B)** the structure of the two haplotypes of the *A. littoralis* cp genome.


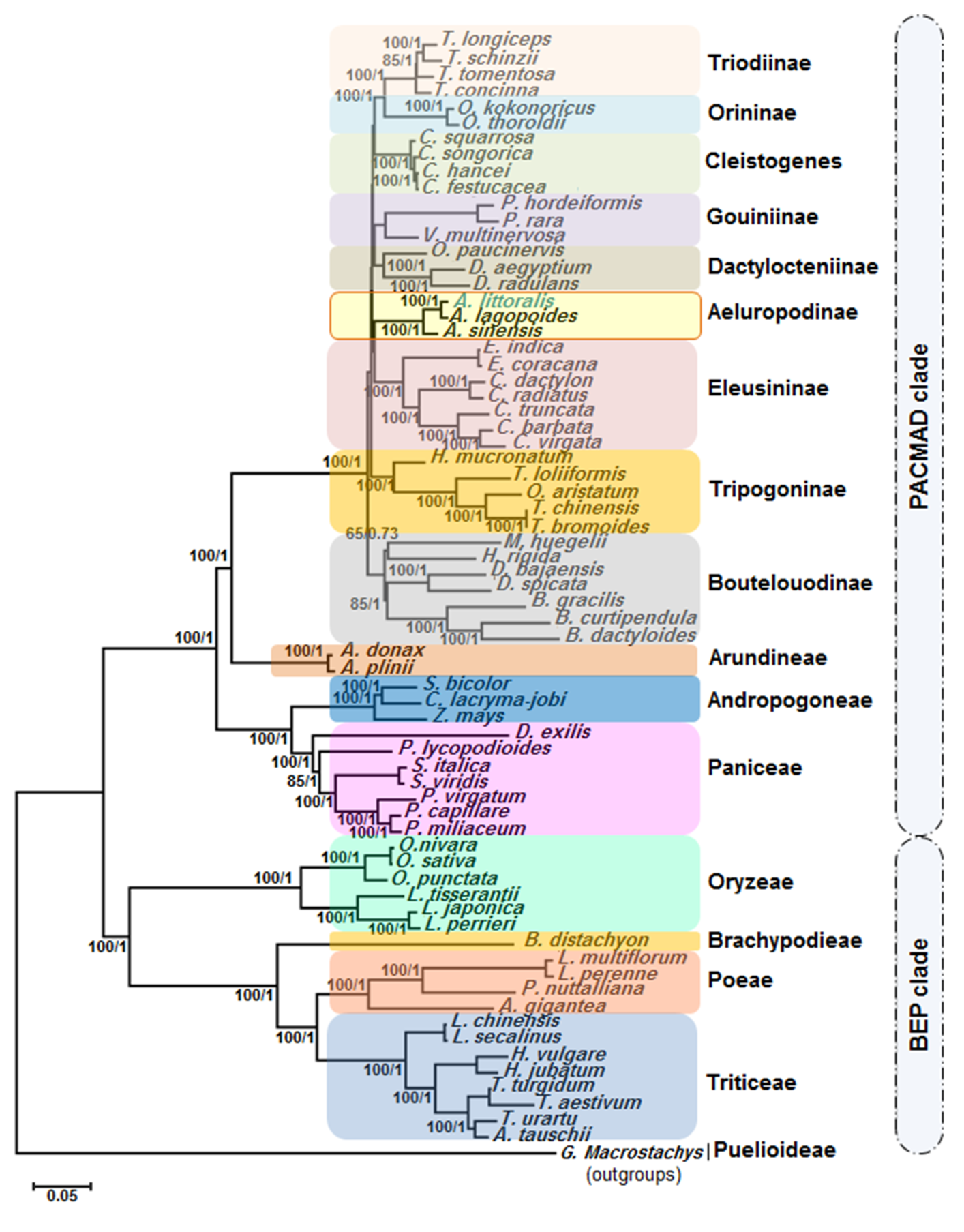


**Figure S2.** Maximum likelihood and Bayesian inference phylogenetic tree based on cp protein sequences of *A. littoralis* and related-species within the Poaceae family. Bootstrap and posterior probability support values are indicated above each node.


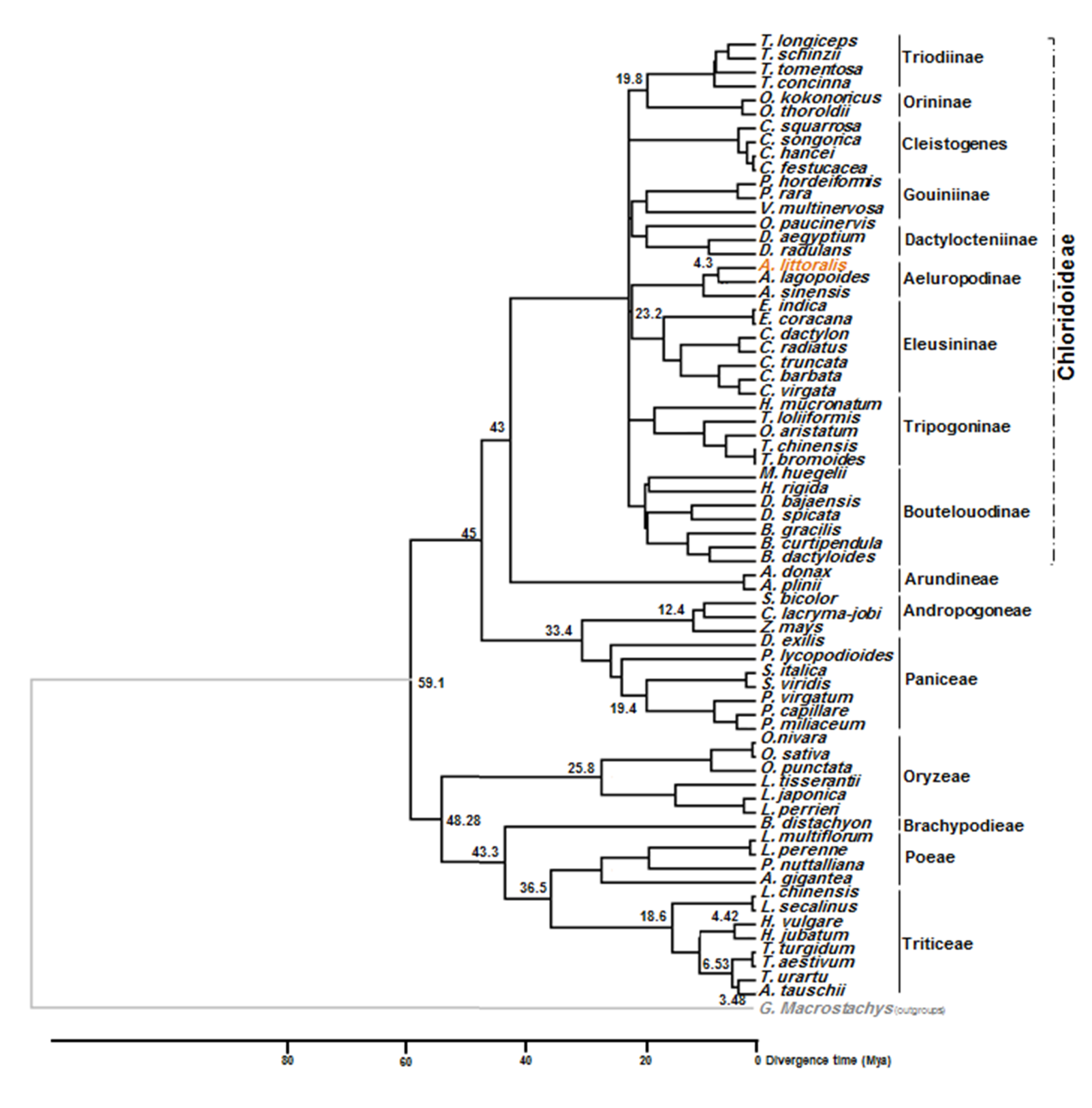


**Figure S3.** Chronogram of *Aeluropus littoralis* and sixty-nine related Poaceae species based on the complete chloroplast genomes inferred by using Mega11. The values represent the estimated divergence time of branching points.
