# Supplementary material for: The newly assembled chloroplast genome of Aeluropus littoralis: molecular feature characterization and phylogenetic analysis with related species
Source: Sci Rep. 2024 Mar 18;14:6472. doi: 10.1038/s41598-024-57141-8 (PMC10948853; doi:10.1038/s41598-024-57141-8)
Supplement: Supplementary file 3 — Supplementary Tables. [file 41598_2024_57141_MOESM3_ESM.docx]

| Genes | Location | Start | End | Direction | Exons | Introns |
| --- | --- | --- | --- | --- | --- | --- |
| *pafI (ycf3)* | LSC | 42646 | 44640 | - | 3 | 2 |
| *rps12* | LSC-IR | 67838 | 89335 | - | 3 | 2 |
| *atpF* | LSC | 33483 | 34874 | + | 2 | 1 |
| *ndhA* | SSC | 111252 | 113391 | - | 2 | 1 |
| *ndhB* | IR | 86267 | 88511 | - | 2 | 1 |
| *ndhB* | IR | 127945 | 130189 | + | 2 | 1 |
| *petB* | LSC | 71573 | 73010 | + | 2 | 1 |
| *petD* | LSC | 73210 | 74437 | + | 2 | 1 |
| *rpl16* | LSC | 78002 | 79493 | - | 2 | 1 |
| *rpl2* | IR | 81443 | 82927 | - | 2 | 1 |
| *rpl2* | IR | 133229 | 133510 | + | 2 | 1 |
| *rps12* | IR | 126321 | 127121 | + | 2 | 1 |
| *rps16* | LSC | 4536 | 5631 | - | 2 | 1 |
| *tRNA-Ala* | IR | 94819 | 95701 | + | 2 | 1 |
| *tRNA-Ala* | IR | 120755 | 121637 | - | 2 | 1 |
| *tRNA-Gly* | LSC | 13092 | 13844 | - | 2 | 1 |
| *tRNA-Ile* | IR | 93966 | 94753 | + | 2 | 1 |
| *tRNA-Ile* | IR | 121703 | 122490 | - | 2 | 1 |
| *tRNA-Leu* | LSC | 47398 | 48032 | + | 2 | 1 |
| *tRNA-Lys* | LSC | 1451 | 3989 | - | 2 | 1 |
| *tRNA-Val* | LSC | 51601 | 52278 | - | 2 | 1 |

**Table S2**. Structure organization of interrupted genes in *A. littoralis* complete cp genome.

| Indices | Location | Consensus Size | Copy Number | Score |
| --- | --- | --- | --- | --- |
| 14855—14981 | Intergenic | 67 | 1.9 | 173 |
| 17625—17669 | intergenic | 22 | 2.0 | 81 |
| 18128—18168 | Intergenic | 20 | 2.0 | 82 |
| 18944—19010 | Intergenic | 35 | 1.9 | 116 |
| 25384—25423 | Intergenic | 21 | 1.9 | 80 |
| 27412—27534 | *rpoC2* | 42 | 3.0 | 162 |
| 27443—27566 | *rpoC2* | 21 | 5.9 | 110 |
| 45427—45470 | Intergenic | 22 | 2.0 | 88 |
| 65971—66091 | *Rps18* | 21 | 5.8 | 170 |
| 112482—112537 | Intergenic | 28 | 2.0 | 103 |

**Table S3.** Predicted long repeats in the *A. littoralis* cp genome

| **Gene** | **N of sites** | **E-value** | **Triplet position** | | **Base editing** | **Aa change** | **Count** |
| --- | --- | --- | --- | --- | --- | --- | --- |
|  |  |  | **First** | **second** |  |  |  |
| ***atpA*** | 2 | 4.00E-03 | — | 2 | C→U | P→L | 1 |
|  |  |  |  |  |  | S→L | 1 |
| ***atpB*** | 1 | 4.00E-12 |  | 1 | C→U | A→V | 1 |
| ***cemA*** | 3 | 1.00E-147 | 2 | 1 | C→U | L→F | 1 |
|  |  |  |  |  |  | P→L | 1 |
|  |  |  |  |  |  | P→S | 1 |
| ***clpP*** | 1 | 1.00E-140 | — | 1 | C→U | P→L | 1 |
| ***matK*** | 9 | 2.00E-20 | 4 | 5 | C→U | A→V | 2 |
|  |  |  |  |  |  | H→Y | 2 |
|  |  |  |  |  |  | L→F | 1 |
|  |  |  |  |  |  | P→L | 1 |
|  |  |  |  |  |  | R→C | 1 |
|  |  |  |  |  |  | S→F | 1 |
|  |  |  |  |  |  | T→I | 1 |
| ***ndhA*** | 6 | 6.00E-105 | 1 | 5 | C→U | S→L | 3 |
|  |  |  |  |  |  | A→V | 1 |
|  |  |  |  |  |  | L→F | 1 |
|  |  |  |  |  |  | S→F | 1 |
| ***ndhB*** | 7 | 2.00E-160 | 1 | 6 | C→U | S→L | 3 |
|  |  |  |  |  |  | P→L | 3 |
|  |  |  |  |  |  | H→Y | 1 |
| ***ndhD*** | 2 | 1.00E-27 | — | 2 | C→U | A→V | 1 |
|  |  |  |  |  |  | S→L | 1 |
| ***ndhF*** | 5 | 2.00E-42 | 2 | 3 | C→U | S→L | 2 |
|  |  |  |  |  |  | P→S | 1 |
|  |  |  |  |  |  | L→F | 1 |
|  |  |  |  |  |  | T→I | 1 |
| ***ndhG*** | 1 | 2.00E-106 | — | 1 | C→U | S→L | 1 |
| ***ndhH*** | 2 | 3.00E-100 | 1 | 1 | C→U | A→V | 1 |
|  |  |  |  |  |  | P→S | 1 |
| ***ndhK*** | 1 | 3.00E-141 | 1 | — | C→U | P→S | 1 |
| ***petB*** | 1 | 8.00E-150 | — | 1 | C→U | P→L | 1 |
| ***psaA*** | 1 | 5.00E-82 | 1 | — | C→U | P→S | 1 |
| ***psbC*** | 1 | 4.00E-06 | 1 | — | C→U | P→S | 1 |
| ***psbD*** | 1 | 2.00E-151 | — | 1 | C→U | A→V | 1 |
| ***psbE*** | 1 | 8.00E-48 | 1 | — | C→U | L→F | 1 |
| ***rbcL*** | 1 | 1.00E-37 | — | 1 | C→U | P→L | 1 |
| ***rpl20*** | 1 | 8.00E-66 | — | 1 | C→U | T→I | 1 |
| ***rpl22*** | 1 | 3.00E-85 | 1 | — | C→U | P→S | 1 |
| ***rpl23*** | 2 | 5.00E-30 | — | 2 | C→U | S→F | 1 |
|  |  |  |  |  |  | T→I | 1 |
| ***rpoB*** | 5 | 6.00E-25 | 1 | 4 | C→U | S→L | 3 |
|  |  |  |  |  |  | P→L | 1 |
|  |  |  |  |  |  | L→F | 1 |
| ***rpoC1*** | 1 | 1.00E-22 | — | 1 | C→U | T→I | 1 |
| ***rpoC2*** | 12 | 6E-18 | 1 | 11 | C→U | S→L | 4 |
|  |  |  |  |  |  | T→I | 2 |
|  |  |  |  |  |  | A→V | 2 |
|  |  |  |  |  |  | P→L | 2 |
|  |  |  |  |  |  | L→F | 1 |
|  |  |  |  |  |  | S→F | 1 |
| ***rps14*** | 1 | 3.00E-59 | — | 1 | C→U | S→L | 1 |
| ***rps18*** | 2 | 5.00E-96 | 1 | 1 | C→U | P→S | 1 |
|  |  |  |  |  |  | S→F | 1 |
| ***rps19*** | 2 | 7.00E-53 | — | 2 | C→U | T→I | 1 |
| ***rps2*** | 1 | 2.00E-147 | — | 1 | C→U | T→I | 1 |
| ***rps8*** | 1 | 2.00E-79 | — | 1 | C→U | S→L | 1 |
| ***ycf2*** | 1 | 2.00E-149 | — | 1 | C→U | S→F | 1 |
| ***ycf3*** | 2 | 2.00E-43 | — | 2 | C→U | T→M | 1 |
|  |  |  |  |  |  | S→F | 1 |

**Table S4.** Predicted RNA editing sites in the A. littoralis cp genome

|  | *Aeluropus littoralis* | *Aeluropus lagopoides* | *Aeluropus sinensis* |
| --- | --- | --- | --- |
| Accession | ON357749 | NC_042858 | NC_060389 |
| Total size (bp) | 135,532 | 135,518 | 135,563 |
| LSC size (bp) | 80,823 | 80,813 | 80,855 |
| SSC size (bp) | 12,691 | 12,685 | 12,688 |
| IR size (bp) | 21,012 | 21,010 | 21,010 |
| Total GC content (%) | 38.24 | 38.22 | 38.27 |
| Number of genes | 133 | 133 | 131 |
| Number of protein-coding genes | 87 | 85 | 85 |
| Number of tRNA genes | 38 | 40 | 38 |
| Number of rRNA genes | 8 | 8 | 8 |

**Table S5.** Comparison of chloroplast genomes features of the genus *Aeluropus*

| Scientific name | Abbreviation | Accession | Clade | subtribe |
| --- | --- | --- | --- | --- |
| *Aeluropus lagopoides* | *A. lagopoides* | NC_042858 | PACMAD | Aeluropodinae |
| *Aeluropus littoralis* | *A. littoralis* | ON357749 |  |  |
| *Aeluropus sinensis* | *A. sinensis* | NC_060389 |  |  |
| *Bouteloua curtipendula* | *B. curtipendula* | NC_029414 | PACMAD | Boutelouinae |
| *Bouteloua dactyloides* | *B. dactyloides* | OM307669 |  |  |
| *Bouteloua gracilis* | *B. gracilis* | NC_029892 |  |  |
| *Hilaria rigida* | *H. rigida* | NC_029896 |  |  |
| *Distichlis bajaensis* | *D. bajaensis* | NC_029894 |  |  |
| *Distichlis spicata* | *D. spicata* | NC_029895 |  |  |
| *Muhlenbergia huegelii* | *M. huegelii* | OM307681 |  |  |
| *Dactyloctenium aegyptium* | *D. aegyptium* | NC_036714 | PACMAD | Dactylocteniinae |
| *Dactyloctenium radulans* | *D. radulans* | NC_042838 |  |  |
| *Odyssea paucinervis* | *O. paucinervis* | MW014308 |  |  |
| *Chloris barbata* | *C. barbata* | NC_029893 | PACMAD | Eleusininae |
| *Chloris truncata* | *C. truncata* | NC 032033 |  |  |
| *Chloris virgata* | *C. virgata* | NC_032034 |  |  |
| *Cynodon dactylon* | *C. dactylon* | NC_034680 |  |  |
| *Cynodon radiatus* | *C. radiatus* | NC_068110 |  |  |
| *Eleusine coracana* | *E. coracana* | MW262987 |  |  |
| *Eleusine indica* | *E. indica* | NC_030486 |  |  |
| *Cleistogenes festucacea* | *C. festucacea* | NC 060392 | PACMAD | Cleistogeninae |
| *Cleistogenes hancei* | *C. hancei* | NC_060395 |  |  |
| *Cleistogenes songorica* | *C.s songorica* | NC_060397 |  |  |
| *Cleistogenes squarrosa* | *C.squarrosa* | NC 060398 |  |  |
| *Orinus kokonoricus* | *O. kokonoricus* | NC_042859 | PACMAD | Orininae |
| *Orinus thoroldii* | *O. thoroldii* | MW057690 |  |  |
| *Perotis hordeiformis* | *P. hordeiformis* | NC_068121 | PACMAD | Gouiniinae |
| *Perotis rara* | *P. rara* | NC_068123 |  |  |
| *Vaseyochloa multinervosa* | *V. multinervosa* | NC 036710 |  |  |
| *Triodia concinna* | *T. concinna* | NC_042862 | PACMAD | Triodiinae |
| *Triodia longiceps* | *T. longiceps* | NC_042864 |  |  |
| *Triodia schinzii* | *T. schinzii* | NC_042870 |  |  |
| *Triodia tomentosa* | *T. tomentosa* | NC_042868 |  |  |
| *Halopyrum mucronatum* | *H. mucronatum* | NC_036688 | PACMAD | Tripogoninae |
| *Oropetium aristatum* | *O. aristatum* | NC_036697 |  |  |
| *Tripogon bromoides* | *T. bromoides* | NC_060399 |  |  |
| *Tripogon chinensis* | *T. chinensis* | NC_060400 |  |  |
| *Tripogonella loliiformis* | *T. loliiformis* | NC_042840 |  |  |
| *Digitaria exilis* | *D. exilis* | NC_024176 | PACMAD | Paniceae |
| *Panicum capillare* | *P. capillare* | NC_030493 |  |  |
| *Panicum lycopodioides* | *P. lycopodioides* | NC_036121 |  |  |
| *Panicum miliaceum* | *P. miliaceum* | NC_029732 |  |  |
| *Panicum virgatum* | *P. virgatum* | NC_015990 |  |  |
| *Setaria italica* | *S. italica* | NC_022850 |  |  |
| *Setaria viridis* | *S. viridis* | NC_028075 |  |  |
| *Coix lacryma-jobi* | *C. lacryma-jobi* | KY596160 | PACMAD | Andropogoneae |
| *Sorghum bicolor* | *S. bicolor* | NC_008602 |  |  |
| *Zea mays* | *Z. mays* | NC_001666 |  |  |
| *Arundo donax* | *A. donax* | NC_037077 | PACMAD | Arundineae |
| *Arundo plinii* | *A. plinii* | NC_034652 |  |  |
| *Leersia japonica* | *L. japonica* | NC_034766 | BEP | Oryzeae |
| *Leersia perrieri* | *L. perrieri* | NC_044486 |  |  |
| *Leersia tisserantii* | *L. tisserantii* | NC_016677 |  |  |
| *Oryza nivara* | *O. nivara* | NC_005973 |  |  |
| *Oryza punctata* | *O. punctata* | NC_027676 |  |  |
| *Oryza sativa* | *O. sativa* | NC_031333 |  |  |
| *Agrostis gigantea* | *A. gigantea* | NC_037162 | BEP | Poeae |
| *Lolium multiflorum* | *L. multiflorum* | NC_019651 |  |  |
| *Lolium perenne* | *L. perenne* | NC_009950 |  |  |
| *Puccinellia nuttalliana* | *P. nuttalliana* | NC_027485 |  |  |
| *Hordeum jubatum* | *H. jubatum* | NC_027476 | BEP | Triticeae |
| *Hordeum vulgare* | *H. vulgare* | NC_008590 |  |  |
| *Leymus chinensis* | *L. chinensis* | NC_044900 |  |  |
| *Leymus secalinus* | *L. secalinus* | MZ595321 |  |  |
| *Triticum aestivum* | *T. aestivum* | NC_002762 |  |  |
| *Triticum turgidum* | *T. turgidum* | LC377262 |  |  |
| *Triticum urartu* | *T. urartu* | NC_021762 |  |  |
| *Aegilops tauschii* | *A. tauschii* | NC_022133 |  |  |
| *Brachypodium distachyon* | *B. distachyon* | NC_011032 | BEP | Brachypodiinae |
| *Guaduella macrostachys* | *G. macrostachys* | NC_061343 | BEP | Puelioideae (Outgroups) |

**Table S6.** List of chloroplast genomes used for phylogenetic analysis
